# Exploring the impact of social influence on parents’ intention to use institutional childcare: a cross-sectional study

**DOI:** 10.1186/s12913-025-12362-z

**Published:** 2025-02-03

**Authors:** Bo Yu, Difan Zhang, Yin Sun, Yuna Yao, Yingji Li, Huiling Hu

**Affiliations:** 1 School of Humanities and Management, Yunnan University of Chinese Medicine, Kunming, 650500 China; 2 Faculty of Management and Economics, Kunming University of Science and Technology, Kunming, 650500 China; 3 College of Preschool Education, Shandong Ying Cai University, Jinan, 250104 China; 4 Institute of Creative Design and Management, National Taipei University of Business, Taipei, 100025 Taiwan

**Keywords:** Intention to use institutional childcare, Social influence, PLS-SEM

## Abstract

**Background:**

China is actively responding to low birth rates by developing childcare institutions to alleviate the parenting pressures on families. However, despite strong childcare demand, the enrollment rate remains low. Few studies have explored the role of social influence in parents' childcare decisions. Therefore, this study will construct a research model to explore the mechanisms of social influence on parents' intentions to use childcare institutions.

**Methods:**

This study used parents of infants and toddlers in mainland China as the study population. Questionnaires were collected through electronic surveys created on a professional website, with 554 responses gathered through purposive sampling. The data were analyzed using Partial Least Squares Structural Equation Modeling (PLS-SEM) and Importance-Performance Map Analysis (IPMA).

**Results:**

The results indicate that perceived benefits are the significant driving variable for parents' intention to use childcare institutions, while price sensitivity and perceived risk are significant barriers. Normative influence and perceived childcare policy significantly affect perceived benefits and price sensitivity, while informational influence has a significant positive effect on perceived risk. Additionally, the Importance-Performance Map Analysis (IPMA) shows that improvements should focus more on price sensitivity given the current low performance.

**Conclusions:**

These findings provide insights for childcare policymakers and institutional childcare managers and will generate a management basis for developing China’s childcare service industry.

**Supplementary Information:**

The online version contains supplementary material available at 10.1186/s12913-025-12362-z.

## Background

In 2023, China's total fertility rate had dropped to just 1.0, raising concerns about low fertility intentions. Low fertility rates lead to a lack of labor pools, increased labor costs, and an aging population, putting tremendous pressure on public finances and social security and hindering socioeconomic development [1]. Low fertility rates arise from many factors, with parenting stress being a significant one. Parenting stress is a pressure that parents feel daily from within the parent–child system. This stress is primarily a result of negative emotions and states that arise from the inability of available social support resources to meet parenting needs [2].

Researchers have a consensus that social support is essential in relieving parenting stress [2–5]. Two sources of social support exist in China: one from grandparents and other relatives, also known as informal support, and the other is public childcare support, also known as formal support. With the gradual liberalization of China’s population birth policy, families’ demand for childcare institution services has risen. Although China’s public childcare services have a promising future, their development still has many problems. On the one hand, private childcare providers have significant investments, high costs, and low input–output ratios, making their survival difficult [6].

On the other hand, although the demand for childcare is strong, it is difficult for nurseries to recruit infants and toddlers, and the vacancy rate of childcare spaces is high [6]. Parents are still concerned about institutional childcare’s cost, safety, and risk. Therefore, it is necessary to gain insight into the demand for childcare services and the factors that influence it among parents in China to identify solutions to promote the development of childcare services and to help policy and decision-makers understand and develop the childcare service industry.

Prior research has been conducted on parental childcare behavior, which can be divided into two main perspectives. From an economic perspective, the influence of parents’ income, the cost of childcare services, the benefits of childcare, and government subsidies on childcare behavior has been explored [7]. On the other hand, from a sociological perspective, it was verified that parents’ childcare behaviors are influenced by their traits and the external environment. For example, Fagnani [8] argued that the differences in mothers' employment aspirations and public attitudes toward childcare are the main reasons for childcare disparity between France and Germany. Zhang et al. [9] found that parents in Bangladesh value the social image of childcare institutions, childcare experience, and perceived value. These factors can help them reduce perceived risks. The above studies have examined childcare behavior and its influencing factors in regions where childcare services are relatively well-developed from different perspectives. However, for regions where childcare services are lagging, it is more important to focus on the study of childcare intention to predict the demand for childcare and thus help childcare service practitioners make the proper judgment. At the same time, when parents make childcare decisions, they are subject to social influences from different sources, which may change their perception of the potential risks/benefits of institutional childcare services and thus influence their intentions to use institutional childcare.

Therefore, this study aimed to explore Chinese parents' intention to use institutional childcare and the associated influencing factors. The research questions are outlined below:

- Construct a model based on integrative Social influence to explore the factors related to the intention to use institutional childcare.
- Examine the associations between the Chinese parents’ intention to use institutional childcare, normative influence, informational influence, childcare policy, perceived risk, perceived benefits, and price sensitivity.
- Rank the significant influencing factors by importance and performance.

The first section of this study is a comprehensive literature review, followed by the construction of the conceptual model. The methodology section describes the research design, including data collection and processing methods. The results section presents the analysis results of the structural equation model. The conclusion emphasizes the main findings and significant contributions of this research.

## Conceptual model

### Social influence

Social influence is a universal phenomenon in human social interaction. Social influence refers to changes in individuals’ behavior, attitudes, and emotions due to interactions with others or groups [10]. Deutsch and Gerard [11] developed a dual process model to divide social influence into normative and informational influence. Normative influence (NI) refers to the influence of conforming to the positive expectations of others by conforming to others or the group to gain approval. This influence affects an individual’s attitudes, norms, and values. Several classical research models, such as the Theory of Planned Behavior (TPB) and Values-Beliefs-Norms Theory (VBN), have identified NI as a significant influence and are widely used in research on human social behavior. Informational influence (II) refers to the impact of individuals receiving information from others as evidence of reality. With the development of computer network technology, II has been more often applied to studying the behavioral decisions of new media users, such as online communities and social media. Moreover, French and Raven [12] regarded social influence as social power and five types of power are classified: reward power, coercive power, statutory power, reference power, and expert power. The study emphasizes the relationship between social influence and power, revealing that laws, regulations, and policies constrain and motivate people. The laws, regulations, and policies related to childcare pursued in China at this stage can be mainly divided into management and normative policies for childcare institutions and subsidized preferential policies for childcare institutions or parents, corresponding to the powers mentioned above as statutory and rewarding powers. The influence of these two types of power can explain parents’ perceptions of childcare policies. Therefore, this study will use the three variables of normative, informational, and policy influence to explain the mechanisms of social influence on parents’ IUIC for infants and toddlers in China.

### Normative influence and perceived risk

NI is a social factor that can be divided into subjective norms (i.e., what significant others think the person ought to do) and descriptive norms (i.e., what significant others themselves do) [13]. In this study, subjective norms refer to parents of young children who are influenced by significant others (e.g., elders, leaders, friends, and childcare experts) to decide whether they should use institutional childcare services. Descriptive norms refer to whether parents of young children perceive that others facing the same childcare difficulties would use institutional childcare services. They have been confirmed as important predictors of PR. Unlike general product consumption, risks arising from institutional childcare services are primarily a potential threat to the health and safety of young children. Parents are highly concerned about the health and safety of their children who are separated from them [14]. The experiences or perspectives of those around can alleviate this concern. For example, Wang [15] argued that the approval of significant others negatively affects one’s level of PR, i.e., NI negatively affects PR. Thus:

H1: NI negatively and significantly affects the PR of Chinese parents toward childcare provider services.

### Informational influence and perceived risk

Distinct from NI, II is a process of internalization by enhancing an individual’s knowledge of his or her environment [11]; therefore, II is related to knowledge. II is divided into mass media and social media, depending on how individuals receive information. Personal influence is no longer influenced by space and personal social circles than in the past, and through networks, personal influence is broader and stronger [16]. II may enhance individuals’ judgments about social and personal risks, such as health risks [17], disaster recovery [18], and new coronavirus prevention behaviors [19]. In addition, Huang [20] found that the frequency of reporting on air pollution information by MM and SM is positively correlated with the public's perceived health risks in his study on air pollution. Thus:

H2: II positively and significantly affects the PR of Chinese parents toward institutional childcare services.

### Perceived childcare policy and perceived risk

Policy influence has been relatively less mentioned in social influence studies. However, policies and regulations have guiding, motivating, and constraining effects that regulate individual behaviors and induce them to develop in the desired direction [21]. For example, in studies of green consumption behavior, policies have been shown to promote consumers’ willingness to consume green [22]. Related studies have demonstrated that government childcare policies can influence parental public childcare behavior, including financial subsidy policies [23] and childcare guarantee policies [24]. In addition, van Dongen et al. [25] found that a lack of awareness of government policies may enhance the perception of health risks and influence the views on electromagnetic field risks among Dutch residents. Thus:

H3: The PCP negatively and significantly affects the PR of childcare provider services among Chinese parents.

### Normative influence and perceived benefits

PB is the extent to which a consumer believes he will benefit from the product or service he is purchasing and is a key part of consumer choice [26]. For parents, childcare services can relieve parental stress and increase employment opportunities for mothers [24], and for children, quality childcare services positively affect healthy child development [27]. Previous research has found that when NI is strong, those who believe the behavior will result in many personal benefits will engage in the behavior more frequently. In other words, the impact of NI on behavior must be assessed based on the individual’s perception of whether the behavior is likely beneficial [28]. In addition, Rimal et al. [29] experimented with participating in health promotion activities (yoga) among American college students to verify the significant positive impact of NI on PB. Thus:

H4: NI positively and significantly affects the PB of childcare provider services for Chinese parents.

### Informational influence and perceived benefits

II affects consumers' decision-making process regarding product evaluation and, thus, consumption intentions [30]. High-quality information can enhance individuals' trust in the information, thereby increasing their PB [31]. However, receiving negative information reports can lower their PB. Notably, II in this study focuses on adverse reports against childcare providers, as the media and public opinion are more likely to notice negative information. Liu and Yang [32] found that media attention (i.e., mass media) and online discussions (i.e., social media) about vaccine scandals reduced parents’ PB of vaccinating their children in a study of Chinese parents’ vaccination intentions. Thus:

H5: II negatively and significantly affects Chinese parents’ PB of institutional childcare services.

### Perceived childcare policy and perceived benefits

The existence of policies may change social norms and influence the attitudes of the public [33]. If a policy provides support and incentives for certain public services, the public may be more inclined to participate, perceiving more excellent benefits [34]. For example, Ma, Wang [35] found that perceived policy tools have a significant positive impact on the PB of waste classification among the rural population in China. Panyagometh and Bian [36] research on the consumption of new energy vehicles in China found that consumption promotion policies influence potential consumers' purchase intentions through PB. Therefore, parents’ perceptions of childcare policies will also positively impact the PB of institutional childcare. Thus:

H6: The PCP positively and significantly affects Chinese parents' PB of childcare provider services.

### Normative influence and price sensitivity

In economics research, price elasticity is often used to explain the "aggregate response" of group consumers to prices at the market level [37]. In marketing management, scholars have used PS to measure differences in individual consumers’ responses to price levels and price changes [37]. PS in this study refers to the perceptions of parents of 0–3 years old of the price level and price changes of institutional childcare services in China. Previous studies have validated the predictors of PS. Parents’ PS to institutional care is reduced due to NI. For example, Sun and Li [38] argue that China is a typical collectivist country that places particular importance on the harmony of identity among groups. Therefore, they will be normatively influenced to purchase green products even though they will pay more when consuming green products. Thus:

H7: NI negatively and significantly affects the PS of Chinese parents to childcare provider services.

### Perceived childcare policy and price sensitivity

Although the direct impact of government policies on consumer behavior is insignificant, they can indirectly influence consumers' purchasing decisions by affecting prices [39]. Policies may influence consumers' emotional preferences, altering their sensitivity to price changes [40]. In addition, Dong et al. [41] studied the effect of consumers’ PS on purchase intention under a subsidy policy in China. They found that policy subsidies brought the price of pure electric vehicles almost within the acceptable price range of consumers, reducing the effect of PS on purchase intentions. Consumers are likely to pay more for a product when they perceive significant benefits for that product [42]. Thus:

H8: PCP negatively and significantly affects the PS of Chinese parents to childcare provider services.

### Perceived benefits and price sensitivity

When consumers perceive significant benefits from a product, they may be willing to pay more, resulting in lower PS [43]. Ku et al. [42] confirmed this in their study on consumers' choices of promotional products, demonstrating that PB has a significant negative impact on PS. In the field of early childhood care and education, the quality of childcare is crucial for child development [44]. Therefore, the quality of childcare services is positively correlated with parents' willingness to pay. Parents are willing to pay a higher price for better childcare services because they believe that good services offer a high value for money [9, 14]. In this way, PB decreases their PS. Thus:

H9: PB negatively and significantly affects the PS of Chinese parents to childcare provider services.

### Perceived risk and intention to use institutional childcare

Behavioral intention is the subjective probability of an individual engaging in a particular behavior [45]. Parents, as consumers, are bound to generate institutional childcare consumption intentions before engaging in childcare behavior, which is essential to any purchase behavior process. By understanding consumers’ purchase intentions and antecedents, decision-makers can develop targeted strategies to improve performance [9]. PR represents a potential behavioral barrier [26]. The greater the PR, the more reluctant an individual will be to generate a purchase and recommend the product or service to others. For example, Marriott and Williams [46] found that PR significantly negatively affects consumers' mobile shopping intention. In addition, Wang et al. [47] research found that PR significantly reduces consumers' intention to use ride-sharing services. Thus:

H10: PR negatively affects Chinese parents’ IUIC.

### Perceived benefits and intention to use institutional childcare

PB is often weighed when consumers make purchase decisions [48]. PB varies according to consumption situations and environments and is a key factor of consumer choice [49]. In consumption behavior research, the impact of PB on behavioral intentions may be assimilated by cognitive factors such as attitudes [50]. Lee [51] explored and integrated various advantages of online banking, forming positive factors known as PB, and empirically tested that PB significantly affects behavioral intentions. This has also been confirmed in the context of online group buying research [52]. Thus:

H11: PB positively and significantly affects the IUIC among Chinese parents.

### Price sensitivity and intention to use institutional childcare

Research has shown that price is a determining factor influencing consumers' behavioral intentions. Differences in the prices of similar products or services can lead to differences in consumers' purchase intentions [53]. As with other service consumption, parental PS influences childcare decisions, especially for low-income families [54]. When the cost of childcare services is high, parents choose to have their care at home (especially mothers) or informal care provided by grandparents, friends, or informal nannies [26]. There is no doubt that sensitivity to different prices may influence consumption intentions. Liang, Choi [53] found that Airbnb consumers are more likely to purchase a cheaper product than a product with the same features and a higher price. Thus:

H12: PS positively and significantly affects Chinese parents’ IUIC.

## Methods

### Data collection

This study used parents of infants and toddlers in mainland China as the study population. Based on 2019–2021 population data from the National Bureau of Statistics of China, the number of newborns in mainland China in the last three years was approximately 37.27 million. Using the product of population size and birth rate, the estimated number of their parents was approximately 74.54 million. Based on Cochran's Formula ($n=\frac{{z}^{2}\text{p}(1-p)}{{e}^{2}})$ [55], the minimum required sample size at a 95% confidence interval (CI) and 5% error is 78. Ethical approval was obtained from the Kunming University of Technology prior to the investigation of this study (approval number: KMUST- MEC-182). Data was collected through purposive sampling on the Wenjuanxing platform (https://www.wjx.cn/). It is China's largest professional online survey platform and provides a paid sample library service. The inclusion criteria were parents of children aged 0–3 years in mainland China. As a result, 586 sample data points were collected from May 2022 to June 2022, and 554 valid responses were collected by excluding the samples with a response time that was too short (less than 100 s) and the same answers. The valid questionnaire recovery rate was 94.54%.

### Measurement instrument

**Fig. 1:**
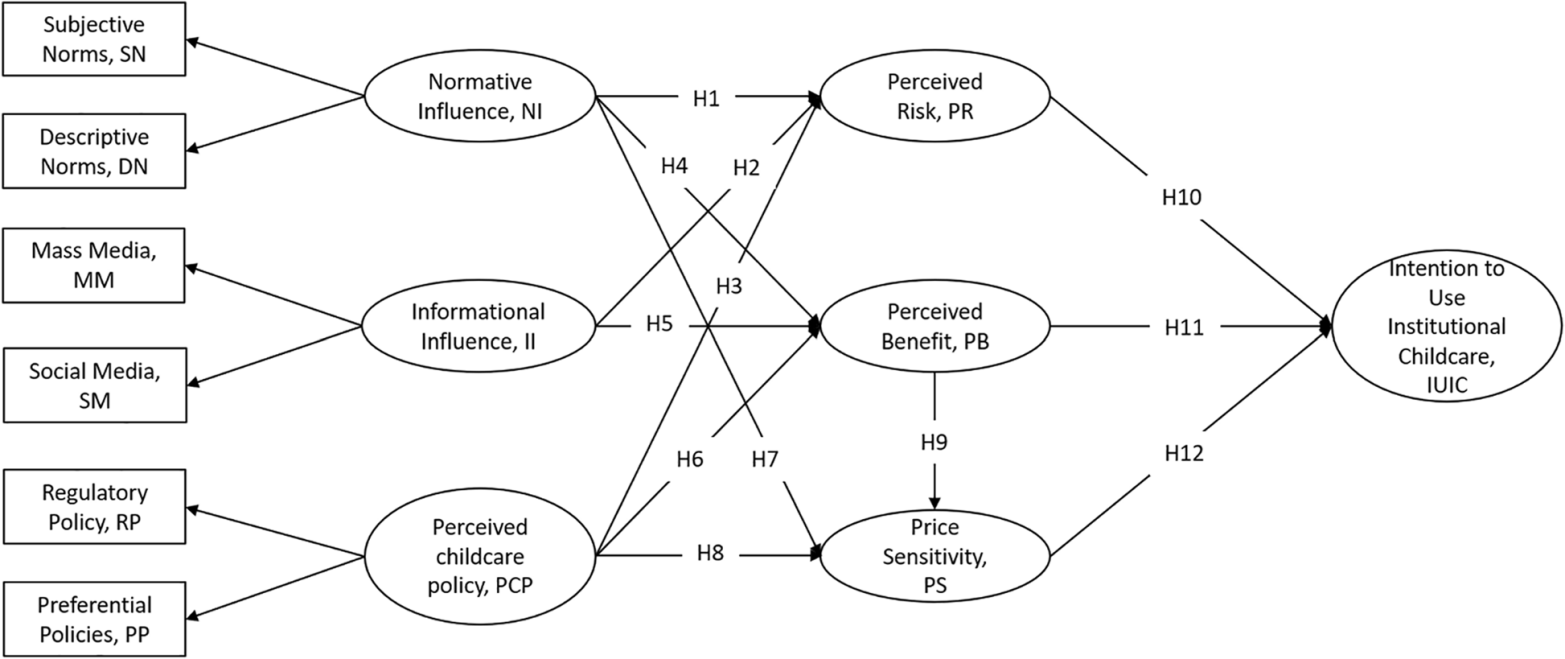
Research model

To predict the most significant factors influencing parents' IUIC, this study developed a comprehensive questionnaire. Hair et al. [56] point out that it is relatively easy to collect large-scale data samples through surveys, which is particularly important for statistical inference. Large sample data can enhance the external validity and generalizability of the research. Therefore, this study uses survey data to validate the hypotheses. The questionnaire contains two sections. The first section is the demographic variables of the respondents, including gender, age, parent’s education, occupation, monthly income, city, childcare status, availability of childcare institutions near their residence, and willingness to pay for care and services. The second section shows the structure of the measurement model (as seen in Fig. 1). Seven core latent variables are involved in this study: NI, II, PCP, PR, PB, PS, and IUIC. Considering the similarity of the research context, the widespread citation of the scale, as well as its reliability and validity, we referenced the scales from the researches by Wang et al. [14], Anderson & Agarwal [57], Cheng et al. [18], Wang et al. [58], and Ramirez & Goldsmith [59]. Then we adapted to the specific scenario of this study by interviewing parents of infants and toddlers and seeking expert advice. The specific measurement items can be found in Supplementary 1.

Regarding scale measurement, Hair et al. [56] suggest that the seven-point Likert scale closely resembles continuous scale characteristics and enhances the reliability and validity of questionnaires, making it the ideal choice. Therefore, this study primarily uses the Likert seven-point scale, where “1” represents strongly disagree, “2” represents disagree, “3” represents partially disagree, “4” represents uncertainty, “5” represents partial agreement, “6” represents agreement, and “7” represents strong agreement. There are some reverse questions in the original scale of PS, and this study made appropriate reversals in the data processing to ensure the consistency of the meaning of the questions. However, there are also viewpoints suggesting that when there are more than five options, most respondents cannot distinguish between the choices [60]. Therefore, this will facilitate respondents' understanding of the measurement items and identification of the options when measuring II. We referenced the measurement scale for II from the study by Cheng et al. [18] and used a 5-point Likert scale. The “1” represents never, “2” represents rarely (once every few years), “3” represents occasionally (once a year), “4” represents sometimes (once every few months), and “5” represents often (more than once a month).

## Results

### Descriptive statistical analysis

This study investigated demographic data such as gender, age, parenting status, mother’s occupation, average monthly income of the couple, and city of residence to understand the fundamental status of parents of infants and toddlers in China. Among the respondents, women accounted for the most significant proportion (335, 60.47%). A total of 288 respondents (51.99%) were between 30–40 years old, roughly in line with the overall childbearing age in China. A total of 384 gave birth to one child, accounting for 69.31%. A total of 252 mothers were corporate employees, accounting for 45.49%. A total of 250 respondents, or 45.13%, had an average household income of CNY 5,000–10,000. Most respondents (257, 46.39%) were from average cities (not first-tier cities and provincial capitals). Regarding childcare maintenance, 216 (38.99%) were shared by both parents and 208 (37.55%) by the principal mother.

### Measurement model verification

Before the path verification of the structural model, the reliability and validity of the constructed structural model should be evaluated. This study evaluated the structural model by examining internal reliability, convergent validity, and discriminant validity. First, Cronbach’s α coefficient and Dijkstra-Henseler’s rho A (ρA) were used to verify the internal consistency of the structural model [61]. The Cronbach’s α coefficient of all variables ranged from 0.760 to 0.944, and the rho A coefficient ranged from 0.765 to 0.945, all higher than the recommended value of 0.700 [61]. This indicates that the model has good internal consistency (see Table 1 for the results). Second, composite reliability (CR) and average variance extraction (AVE) were used to evaluate the aggregation validity of the model. The CR value is between 0.893- 0.960, higher than the recommended value of 0.700 [61], and the AVE value is between 0.689- 0.857, both higher than the recommended value of 0.500. Therefore, the model has aggregation validity (as shown in Table 1). Finally, the Fornell-Larcker method is used to test the model’s validity. The square root of the diagonal AVE is larger than the off-diagonal number (as shown in Table 2). The latent variable's factor load is higher than any cross load, indicating the research model's discriminant validity.

**Table 1:** First-order construct reliability and convergent validity

Latent variables	Average	Standard deviation	Cronbach’s α	rho_A	CR	AVE
SN	4.655	1.572	0.924	0.924	0.946	0.814
PP	5.694	1.207	0.824	0.824	0.895	0.740
DN	4.963	1.479	0.762	0.765	0.893	0.807
MM	2.774	0.957	0.859	0.865	0.905	0.704
PB	5.090	1.291	0.812	0.838	0.887	0.724
PS	3.051	1.405	0.760	0.765	0.893	0.806
PR	4.854	1.362	0.909	0.916	0.930	0.689
IUIC	4.994	1.523	0.944	0.945	0.960	0.857
RP	4.813	1.516	0.926	0.926	0.947	0.818
SM	2.774	0.999	0.885	0.887	0.921	0.744

*CR* Composite reliability, *AVE* Average variance extraction, *SN* Subjective norm, *PP* Preferential policies, *DN* Descriptive norms, *MM* Mass media, *PB* Perceived benefits, *PS* Price sensitivity, *PR* Perceived risk, *IUIC* Intention to use institutional childcare, *RP* Regulatory policies, *SM* Social media

**Table 2:** Discriminant validity of first-order constructs

Latent variables	SN	PP	DN	MM	PB	PS	PR	IUIC	RP	SM
SN	0.902									
PP	0.462	0.860								
DN	0.815	0.501	0.899							
MM	−0.118	−0.083	−0.060	0.839						
PB	0.609	0.503	0.577	−0.076	0.851					
PS	−0.614	−0.386	−0.540	0.124	−0.613	0.898				
PR	0.013	0.106	0.098	0.385	0.220	0.128	0.830			
IUIC	0.759	0.448	0.693	−0.156	0.663	0.692	−0.004	0.926		
RP	0.591	0.484	0.536	−0.202	0.398	0.467	−0.109	0.476	0.905	
SM	−0.094	−0.086	−0.051	0.852	−0.077	−0.095	0.381	−0.150	−0.175	0.863

*SN* Subjective norm, *PP* Preferential policies, *DN* Descriptive norms, *MM* Mass media, *PB* Perceived benefits, *PS* Price sensitivity, *PR* Perceived risk, *IUIC* Intention to use institutional childcare, *RP* Regulatory policies, *SM* Social media

### Path analysis

**Fig. 2:**
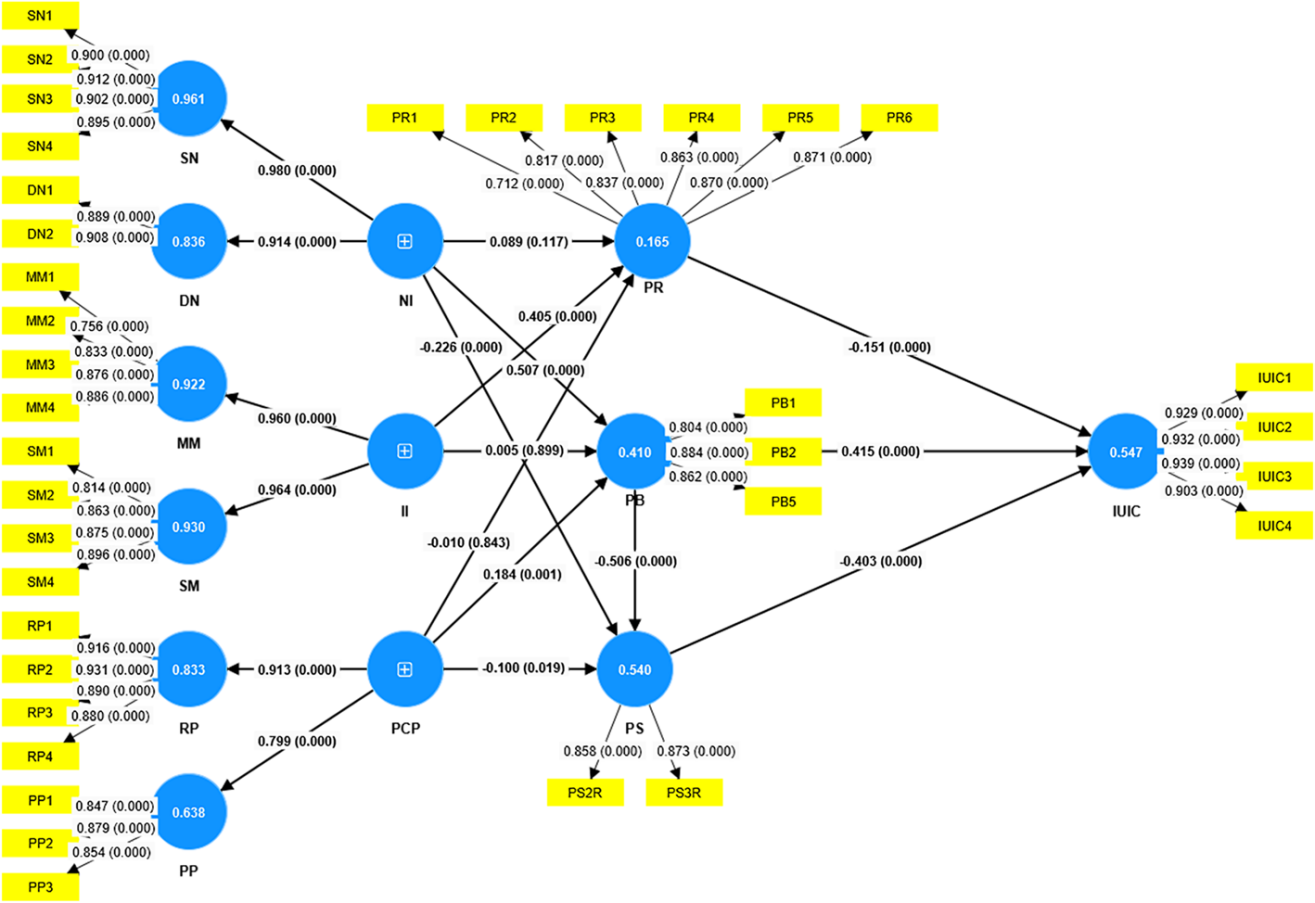
Standardized path coefficient and significance

In this study, Smart PLS 4 was used to validate the structural model. Chin [62] suggested using R^2^ in endogenous structures to evaluate the predictive ability of structural models. The model can explain 54.7% of the variance of the IUIC, which is higher than the recommended value of 30%, indicating that the model’s prediction ability is satisfactory. The results (Table 3) show that NI can significantly affect PB (b = 0.507, *P* < 0.001) and PS (b = −0.226, *P* < 0.001), and H4 and H7 are supported. II could significantly affect PR (b = 0.405, *P* < 0.001), and H2 was supported. PB (b = 0.184, *P* < 0.001) and PS (b = −0.100, *P* = 0.019) were significantly affected by the PCP. H6 and H8 were supported. PB can significantly affect PS (b = −0.506, *P* < 0.001), and H9 is supported. PR (b = −0.151, *P* < 0.001), PB (b = 0.415, *P* < 0.001), and PS (b = −0.403, *P* < 0.001) significantly affected the IUIC, and H10, H11, and H12 were supported. In addition, H1, H3, and H5 were not supported (*P* > 0.05), indicating that normative impact and the perception of nursery policy had no significant impact on PR and informational impact on PB. See Fig. 2 for the regression coefficients of the structural equation model.

**Table 3:** Path analysis

Hypothesis	Path	Original sample (O)	Sample mean (M)	Standard deviation (STDEV)	T statistics (|O/STDEV|)	*P* values
H1	NI—> PR	0.089	0.090	0.057	1.568	0.117
H2	II—> PR	0.405	0.406	0.043	9.417	0.000
H3	PCP—> PR	−0.010	−0.010	0.053	0.198	0.843
H4	NI—> PB	0.507	0.506	0.055	9.291	0.000
H5	II—> PB	0.005	0.005	0.037	0.127	0.899
H6	PCP—> PB	0.184	0.186	0.056	3.268	0.001
H7	NI—> PS	−0.226	−0.225	0.050	4.524	0.000
H8	PCP—> PS	−0.100	−0.101	0.043	2.348	0.019
H9	PB—> PS	−0.506	−0.505	0.042	12.138	0.000
H10	PR—> IUIC	−0.151	−0.150	0.028	5.292	0.000
H11	PB—> IUIC	0.415	0.415	0.049	8.430	0.000
H12	PS—> IUIC	−0.403	−0.404	0.047	8.606	0.000

*O* Original sample, *M* Sample mean, *STDEV* Standard deviation, *NI* Normative influence, *II* Informational influence, *PCP* Perceived childcare policy, *PB* Perceived benefits, *PS* Price sensitivity, *PR* Perceived risk, *IUIC* Intention to use institutional childcare

### Importance-performance map analysis (IPMA)

**Fig. 3:**
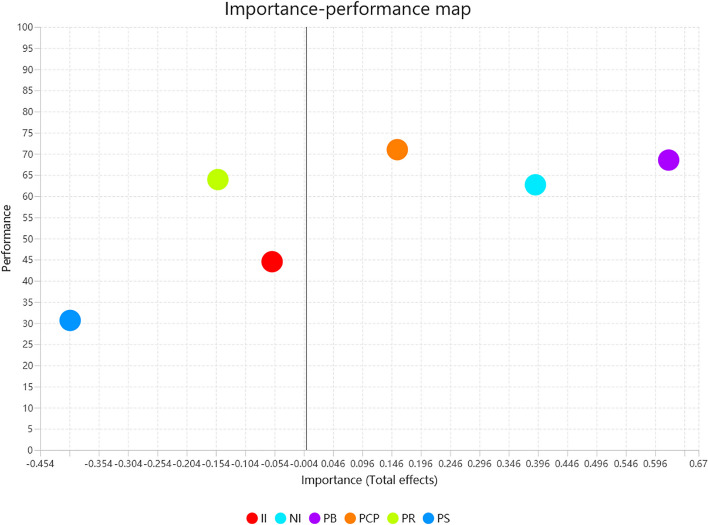
Importance-performance map analysis

IPMA was further performed to identify the most important constructs that needed additional improvement to urge parents to use institutional childcare. The results showed (as shown in Table 4 and Fig. 3) that three structures promote IUIC, while three structures have a suppressive effect. Among them, PB (0.619), PS (−0.403), and NI (0.302) are considered key structures, with their absolute importance exceeding the average absolute value (0.296). However, the performance value of PS (30.633) is not satisfactory, as it is below the average performance value (56.881). Therefore, the IPMA reveals that improvement efforts should focus on PS, as it largely suppresses parents' use of childcare facilities.

**Table 4:** Importance-performance map analysis

Constructs	Importance	Performance
II	−0.058	44.518
NI	0.392	62.691
PB	0.619	68.527
PCP	0.156	70.995
PR	−0.151	63.925
PS	−0.403	30.633
Average absolute value	0.296	56.881

*II* Informational influence, *NI* Normative influence, *PB* Perceived benefits, *PCP* Perceived childcare policy, *PR* Perceived risk, *PS* Price sensitivity

## Discussion

### Key findings

According to the results of the multivariate analysis, there are four key findings.

First, among the three dimensions of social influence, II is a statistically significant driver of PR, which suggests that H2 is supported. This result is consistent with Huang [20] research. With the continuous development and popularization of smartphones and mobile communication technology in China, people can obtain the latest information anytime and anywhere. At the same time, negative information is more likely to attract public attention and spread quickly and widely. As a result, negative stories related to institutional childcare are more likely to attract attention in mass media and social media. Such negative information became the only significant driver of the PR of childcare institutions among Chinese parents. However, the NI and PCP have insignificant adverse effects on PR, and H1 and H3 are unsupported. Moreover, there are differences from the research findings of Wang [15] and van Dongen et al. [25]. The accident risk in care institutions is sporadic, and the experience of others cannot effectively eliminate the risk, so the NI cannot act on the PR. At the same time, although the government has introduced childcare policies, especially regulatory ones, it will take some time to implement them from the national to the regional level. In addition, the sudden closure of recently established government demonstration childcare institutions and non-refundable childcare fees has increased parents' distrust of government policies. So, these policies have not reduced parents' concerns about hidden risks in childcare institutions.

Second, NI and PCP are statistically significant factors influencing PB and PS, while PB also significantly affects PS. Thus, H4, H6, H7, H8 and H9 are supported. Specifically, NI has a significant positive impact on PB in parents' decisions regarding childcare institutions, which is consistent with Rimal et al.’s [29] research findings. Unlike general service consumption, parents attach great importance to the childcare issue, collect relevant information before making childcare decisions, consult the opinions of people around them, and follow most people’s choice of childcare. At the same time, people with institutional care experience are also willing to share their own experiences and perceptions, predominantly positive and good experiences, which positively affects the PB of parents of infants and toddlers. NI significantly reduces PS, consistent with Sun et al.’s [38] research conclusions. This indicates that a good reputation and the choices of those around them can lead parents to overlook the impact of childcare costs. Consistent with Ma et al.'s [35] research findings, PCP significantly impacts PB. The favorable policies introduced by the government have given parents hope for developing the childcare industry, enhancing their trust in childcare institutions. In addition, PCP has a significant negative impact on PS, consistent with Dong et al.'s [41] research findings. This is due to government subsidy policies that alleviate parents' financial burden regarding childcare expenses. However, the negative impact of II on PB is not significant, which suggests that H5 is not supported. This contrasts with Liu et al.’s [63] research findings. This may be because parents are likely to assess the benefits of childcare institutions based on their own or those around them's direct experiences, which are often more convincing than media reports.

Finally, PR, PB, and PS are statistically significant factors influencing parents’ IUIC, which suggests that H10, H11, and H12 are supported. Among them, PR has a significant negative impact on IUIC, which research findings align with Marriott & Williams [46] and are different from Wang et al. [14]. Compared with other studies, the sample size of this study is more prominent, and the sample distribution is relatively extensive. At the same time, since children are the hope of families and belong to vulnerable groups, parents cannot accept any risk of harm to their children, especially when parents themselves cannot accompany them all the time. Therefore, this PR will be infinitely magnified. Therefore, because of these potential risks, parents will choose other childcare modes to replace institutional childcare, thus reducing the IUIC. PB positively and significantly affects the IUIC, which is consistent with the research results of Liu et al. [63].

Furthermore, the IPMA showed that PB was the most important predictor. This shows that parents are motivated to send their children to childcare institutions because they realize that childcare institutions can relieve the pressure of parenting and that the scientific concept of childcare contributes to children’s physical and mental health. PS is also a significant influencing factor of parents’ IUIC, which confirms previous studies [53]. The IUIC still belongs to service consumption intention, which aligns with the general law of consumer behavior. Therefore, when institutional care costs are reduced, parents are encouraged to send their children to institutional care. In contrast, if the cost of institutional care services becomes a significant part of the family’s monthly expenses, especially if it is higher than one parent’s monthly income, parents will choose other methods of childcare to replace institutional care.

### Theoretical implications

This study examined the effect of social influences on parents’ IUIC. Only previous studies have validated the TPB of subjective norms on parents’ IUIC [14]. This study validated the effects of three social, normative, informational, and perceived policies on parents’ IUIC. It expands the literature on the parental use of institutional childcare services by providing additional insight from multiple perspectives. Furthermore, it also allows scholars to extend social influences to other research settings.

Additionally, this study reveals the role of PS in childcare decision-making. Most existing studies have built mathematical models to analyze the impact of childcare service prices on childcare decisions [7], while empirical studies are less common. At the same time, due to the specificity of childcare service recipients, parents’ responses to price can be different from general service consumption, and analysis of parents’ price responses alone often fails to present this complexity. This study, however, provides empirical evidence that PS is an essential antecedent to parents’ institutional childcare decisions and extends the scope of PS research.

### Practical implications

This study suggests three practical implications for childcare policymakers and agency administrators to facilitate the transformation of parents’ potential demand for institutional childcare use into effective IUIC.

First, among social influences, II can hinder parents’ institutional childcare use intentions through PR. Negative information about childcare providers can be quickly and widely disseminated through mass media and social media, amplifying its negative impact. It will be detrimental to the healthy development of the childcare service industry. Therefore, the relevant government administration can eliminate the negative informational impact by selecting model childcare providers and investing in publicity in mass media to establish a positive image of childcare providers. As childcare providers, they can also post videos of the childcare providers’ good childcare environment, disseminate scientific childcare concepts, and engage in fun interactions between caregivers and young children by establishing institutional social media accounts and strengthening their positive publicity, good communication, and interaction with parents, deepening parents’ perceived interest in childcare institutions, reducing the PR caused by lack of understanding, and thus increasing parents’ IUIC.

Second, government departments have issued a series of relevant policies to promote the development of the childcare service industry since 2019. However, these policies mainly focus on supporting and supervising childcare institutions, while subsidies and preferential policies for parents of young children lag. Hence, only practitioners in the childcare industry pay more attention to and understand these policies and parents’ perceptions of childcare policies are generally weak. Therefore, when making policies, the government can pay special attention to the preferential policies of childcare subsidies for parents of young children, reduce the pressure on parents’ childcare costs, enhance parents’ perception of the benefits of childcare institutions, reduce PS, and improve parents’ IUIC. In addition, by installing surveillance cameras in care institutions, parents can learn about their children’s activities anytime and anywhere to effectively supervise care institutions in an all-around and whole-process way. As a result, it improves parents’ PB and reduces PR; at the same time, childcare institutions can also avoid some conflicts and legal disputes.

Third, this study finds that NI impacts the IUIC through PB. Therefore, the NI can be strengthened to promote parents’ IUIC. Currently, a relatively mature professional service consumption evaluation platform has been formed in the fields of online shopping, food consumption, and hotel accommodation consumption in mainland China. Through this service consumption platform, potential consumers can have a relatively comprehensive and objective understanding of the consumer group’s overall evaluation of goods or services. The service consumption evaluation platform will become an essential channel for NI output. Parents can obtain more detailed information about childcare institutions and objective evaluations from peer parents on the platform, which helps parents make wise decisions.

### Limitations and future research

Although this study has verified the impact of social influence on parents’ intention of institutional childcare and has made specific theoretical and practical contributions, it still has certain limitations. First, this study attempted to verify the linear and nonlinear relationships between variables through two methods. The two methods complement each other and improve the accuracy of the results. Future research could employ other approaches, such as follow-up and experimental studies, to examine the causal relationship between these variables. Second, the nonprobability sampling method used in this study may cause some selection bias and cannot fully represent the actual situation of parents in China. In a subsequent study, the probability sampling method of stratified sampling can be adapted further to verify the results in a large population sample. Third, different population groups can be compared; for example, different ages, cities, and family parenting workforces can be used as the criteria to divide the survey objects, and the differences in statistical results may lead to some interesting findings. Fourth, this study examined only the influence of negative information and ignored the positive information about childcare services. Follow-up studies could examine II's positive and negative effects on childcare decisions. Finally, this study only focuses on the influence of Social influence on the IUIC in Chinese parents. Other factors, such as institutional trust, childcare image, service quality, childcare subsidy mechanism, and intergenerational support, are not considered. In future research, a new model can be considered for verification.

## Conclusions

Among social influences, NI and PCP are statistically significant factors driving PB and PS. Additionally, II significantly affects PR. Furthermore, PR, PB, and PS significantly influence parents' IUIC. Focusing on these factors will help childcare policymakers and institutional childcare managers make informed management decisions and promote the sustainable development of China’s childcare service industry.

## Supplementary Information

Supplementary Material 1.

## Data Availability

The datasets used and analysed during the current study are available from the corresponding author on reasonable request.

## Abbreviations

SEM: Structural Equation Modeling
OECD: Organization for Economic Co-operation and Development
SI: Social Influence
TPB: Theory of Planned Behavior
VBN: Values-Beliefs-Norms
NI: Normative Influence
SN: Subjective Norm
DN: Descriptive Norms
II: Informational Influence
MM: Mass Media
SM: Social Media
PCP: Perceived Childcare Policy
PP: Preferential Policies
RP: Regulatory Policies
PR: Perceived Risk
PB: Perceived Benefits
PS: Price Sensitivity
IUIC: Intention to Use Institutional Childcare
CI: Confidence Interval
PLS: Partial Least Squares
ρA: Rho A
CR: Combined Reliability
AVE: Average Variance Extraction
