# Supplementary material for: Exploring the impact of social influence on parents’ intention to use institutional childcare: a cross-sectional study
Source: BMC Health Serv Res. 2025 Feb 3;25:198. doi: 10.1186/s12913-025-12362-z (PMC11792571; doi:10.1186/s12913-025-12362-z)
Supplement: Supplementary file 1 — Supplementary Material 1. [file 12913_2025_12362_MOESM1_ESM.docx]

**Supplementary 1****. Measurement items and sources.**

| Latent variables | Item | Source (s) | Loading |
| --- | --- | --- | --- |
| SN1 | People who significantly influence me (e.g., relatives, elders) think I can send my child to an institutional childcare. | Wang et al. [14] | 0.900 |
| SN2 | Someone I respect (e.g., leader, teacher) thinks I can send my child to an institutional childcare. |  | 0.912 |
| SN3 | A childcare professional I follow thinks I can send my child to an institutional childcare. |  | 0.902 |
| SN4 | My friend suggests that I send my child to an institutional childcare. |  | 0.895 |
| DN1 | I think one of my friends sends his/her child to an institutional childcare. | Anderson & Agarwal [57] | 0.889 |
| DN2 | I think most urban parents send their children to an institutional childcare. |  | 0.908 |
|  | Over the past few years, how often have you seen the following topics in the mass media (e.g., TV, news websites, mobile news apps, etc.)? | Cheng et al. [18] |  |
| MM1 | Institutional childcare closing. |  | 0.756 |
| MM2 | Infectious diseases in institutional childcare (e.g., confirmed cases of new crowns, etc.). |  | 0.833 |
| MM3 | Child abuse at an institutional childcare. |  | 0.876 |
| MM4 | A public health incident at an institutional childcare (e.g., mass vomiting or diarrhea caused by food safety issues). |  | 0.886 |
|  | Over the past few years, how often have you seen the following topics on social media (e.g., Weibo, WeChat, Zhihu, Xiaohongshu, mother and baby forums, etc.)? | Cheng et al. [18] |  |
| SM1 | Institutional childcare closing. |  | 0.814 |
| SM2 | Infectious diseases in institutional childcare (e.g., confirmed cases of new crowns, etc.). |  | 0.863 |
| SM3 | Child abuse at an institutional childcare. |  | 0.875 |
| SM4 | A public health incident at an institutional childcare (e.g., mass vomiting or diarrhea caused by food safety issues). |  | 0.896 |
| RP1 | I think the current governmental standards for institutional childcare are being effectively implemented. | Wang et al. [14] | 0.916 |
| RP2 | I believe the current government regulations for institutional childcare are being effectively enforced. |  | 0.931 |
| RP3 | I believe that the government’s registration and record-keeping of childcare institutions are improving the regulation of childcare institutions. |  | 0.890 |
| RP4 | I think the government is already regulating the whole process of childcare institutions. |  | 0.880 |
| PP1 | Including childcare expenses for children under 3 in the personal income tax deduction policy will make me send the child to an institutional childcare. | Wang et al. [14] | 0.847 |
| PP2 | The government’s financial subsidy for families who enroll their children under 3 in childcare will make me send my child to an institutional childcare. |  | 0.879 |
| PP3 | The government’s price limit on institutional childcare fees will allow me to send my child to an institutional childcare. |  | 0.854 |
| PR1 | Institutional childcare may close if they are not well run and there are no refunds. | Wang et al. [58] | 0.712 |
| PR2 | There is a risk of cross-contamination of diseases (e.g., influenza) at the institutional childcare. |  | 0.817 |
| PR3 | Institutional childcare personnel may abuse young children. |  | 0.837 |
| PR4 | Unreasonable space layouts in institutional childcare may result in accidental injury to young children. |  | 0.863 |
| PR5 | Food safety problems in institutional childcare may lead to intestinal diseases in young children. |  | 0.870 |
| PR6 | Safety accidents may occur due to the negligence of institutional childcare personnel. |  | 0.871 |
| PB1 | Childcare services may alleviate the childcare conflicts that exist in my family. | Wang et al. [58] | 0.804 |
| PB2 | Childcare services may relieve the childcare stress I am currently experiencing. |  | 0.884 |
| PB3 | If I send my child to childcare, I (or my wife) will be able to go back to my workplace with peace of mind. |  | Removed |
| PB4 | The childcare services scientific parenting philosophy would benefit my child’s physical and healthy development. |  | Removed |
| PB5 | The childcare services scientific parenting philosophy is good for my child’s mental health and development. |  | 0.862 |
| PS1 | If I thought childcare services were expensive, I would choose other ways to care for my child. | Ramirez & Goldsmith [59] | Removed |
| PS2(R) | I do not mind spending more money on institutional childcare services for my child. |  | 0.858 |
| PS3(R) | It is worth spending money for my child to get good care and educational guidance in a childcare institution. |  | 0.873 |
| IUIC1 | I would consider sending my child to childcare. | Wang et al. [58] | 0.929 |
| IUIC2 | I will most likely send my child to childcare. |  | 0.932 |
| IUIC3 | I plan to send my child to childcare. |  | 0.939 |
| IUIC4 | I would recommend that a friend or colleague send his or her child to childcare. |  | 0.903 |
